# Modeling the AC Electrokinetic Behavior of Semiconducting Spheres

**DOI:** 10.3390/mi10020100

**Published:** 2019-01-29

**Authors:** Pablo García-Sánchez, Jose Eladio Flores-Mena, Antonio Ramos

**Affiliations:** 1 Departamento de Electrónica y Electromagnetismo, Facultad de Física, Universidad de Sevilla, Avda. Reina Mercedes s/n, 41012 Sevilla, Spain; ramos@us.es; 2 Facultad de Ciencias de la Electrónica, Benemérita Universidad Autónoma de Puebla, Av. San Claudio y 18 Sur, San Manuel, Puebla 72570, Mexico; jefloresmena@gmail.com

**Keywords:** electrokinetics, dielectrophoresis, electrorotation, semiconductors, microspheres

## Abstract

We study theoretically the dielectrophoresis and electrorotation of a semiconducting microsphere immersed in an aqueous electrolyte. To this end, the particle polarizability is calculated from first principles for arbitrary thickness of the Debye layers in liquid and semiconductor. We show that the polarizability dispersion arises from the combination of two relaxation interfacial phenomena: charging of the electrical double layer and the Maxwell–Wagner relaxation. We also calculate the particle polarizability in the limit of thin electrical double layers, which greatly simplifies the analytical calculations. Finally, we show the model predictions for two relevant materials (ZnO and doped silicon) and discuss the limits of validity of the thin double layer approximation.

## 1. Introduction

Manipulation of micro- and nano-particles with electric fields is a topic of research with applications in diverse fields such as biotechnology and microelectronics [1,2]. Recent experimental work has focused on conducting particles, and for example, semiconductor nanowires can be rotated and transported within microchannels with embedded electrodes [3,4,5]. The rotation speed of metal microspheres [6,7,8] and metal nanowires [9,10,11] is controlled by tuning the frequency and amplitude of the externally-applied rotating field. Likewise, the orientation of metal nanowires is achieved upon application of AC signals [12,13,14,15]. Moreover, the electrorotation amplitude of semiconducting nanowires can be modulated by laser illumination [16]. Furthermore, electrical properties of semiconducting microparticles can be characterized on chip [17,18]. Other examples include self-assembly of metal particles induced upon application of AC electric fields [19,20] and electric field-induced motion and assembly of Janus metallodielectric spheres [21,22,23,24,25].

With respect to the theoretical work, much effort has been recently put into the study of the electrokinetic behavior of metal particles in electrolytes [26,27,28,29]. These works show that, in the case of metallic particles, the electrokinetic response is determined by the formation of an induced electrical double layer (EDL) at the interface between the particle and the electrolyte [30,31]. In a few words, the applied electric fields induce the EDL and interact with its charges, generating electrical forces on the particles and electrosmotic flows around them. Despite the increasing number of experimental papers on electrical manipulation of semiconducting microparticles, not much theoretical work has been devoted to them. Recently, we studied the electrorotation and electroorientation of semiconducting nanowires, and numerical results were compared with published experimental data [32]. These numerical results reveal that, for the case of semiconducting particles, two relaxation mechanisms play a role in the polarization of the particle: the charging of the EDL, as described for metallic particles, and the Maxwell–Wagner relaxation, which arises from the contrast in electrical properties between the liquid and the particle.

The goal of the present work is to obtain analytical solutions for the electrokinetic behavior of a spherical semiconducting particle immersed in an electrolyte. We address the general problem for a spherical particle with an arbitrary size with respect to the thickness of the EDL. We also show the solution to the simpler problem in which the particle diameter is much greater, i.e., the thin double layer approximation. We evaluate the analytical expressions for the case of two semiconducting materials commonly used in applications: ZnO and n-type silicon. In experiments, the electrokinetic manipulation of microparticles is usually carried out with microelectrode structures fabricated on glass substrates. Since particles are heavier than water, they rest on the glass substrate when moving under the action of the electric fields. For this reason, and for future comparison with experimental data, we include the Appendix A where we study the wall influence on the electrokinetic motion of the microspheres.

## 2. Theory

**Figure 1:**
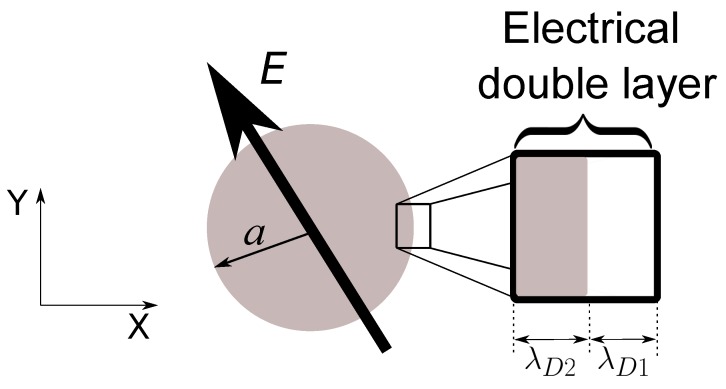
Semiconducting sphere of radius *a* subjected to an AC electric field. Electrical charges are induced at the particle-electrolyte interface giving rise to an electrical double layer (EDL).

Let us consider a spherical particle of semiconducting material with radius *a* and immersed in an aqueous electrolyte (see Figure 1). The particle-electrolyte system is subjected to an AC electric field with angular frequency ω, and as a consequence of the applied field, electric charges are induced at the interface between the particle and the electrolyte. We are interested in the effects of the applied field interacting with these induced electrical charges. In particular, our goal is to calculate the dielectrophoretic (DEP) and electrorotation (ROT) responses.

For the study of the dielectrophoresis, we will assume that the particle-electrolyte system is subjected to a nonuniform electric field of the form E(r,t)=Re[E0(r)exp(iωt)], i.e., the direction of the AC field is constant at each point. Here, Re[⋯] stands for real part of [⋯] and *i* is the imaginary unit. As usual, the point dipole approximation is valid as long as the spherical particles are smaller than the typical length for variation of the electric field. Within this approximation, the time-averaged dielectrophoretic force is obtained as F(DEP)=(1/2)Re[p·∇E*], where * indicates the complex conjugate and p is the induced dipole in the system. On the other hand, in electrorotation experiments, the applied field is of the form E(r,t)=Re[E0(ux−iuy)exp(iωt)], corresponding to a homogeneous electric field within the XY plane with constant magnitude and rotating counterclockwise with angular frequency ω. For this applied field, the time-averaged electrical torque on the system is τ(ROT)=(1/2)Re[p×E*].

The instantaneous induced dipole on the particle can be written as p=Re[p˜exp(iωt)], where $\tilde{p}$ is the dipole phasor and is usually written as a function of the particle polarizability ($\tilde{\alpha }$) as $\tilde{p}=\tilde{\alpha }E=4\pi \varepsilon_{1}a^{3}\tilde{K}\left(\omega \right)E$, where $\varepsilon_{1}$ is the permittivity of the liquid (electrolyte), and we have introduced the Clausius–Mossotti factor $\tilde{K}\left(\omega \right)$. Thus, the DEP force and ROT torque can be written as follows:

(1) F(DEP)=πε1a3Re[K˜(ω)]∇E02

(2) τ(ROT)=−4πε1a3Im[K˜(ω)]E02uz

In the steady state, the electrical force and torque will be balanced, respectively, by the fluid viscous drag and viscous torque on the particle:

(3) $$\begin{array}{ccc} F_{\mathrm{drag}} & = & -6\pi \eta av \end{array}$$

(4) $$\begin{array}{ccc} \tau_{\mathrm{viscous}} & = & -8\pi \eta a^{3}\Omega \end{array}$$

where η is the fluid viscosity and v and Ω are, respectively, the velocity and angular velocity of the sphere. The DEP velocity and ROT angular velocity can be written as:

(5) v(DEP)=ε1a26ηRe[K˜(ω)]∇E02

(6) Ω(ROT)=−ε12ηIm[K˜(ω)]E02uz

Thus, knowledge of the real and imaginary parts of the Clausius–Mossotti factor predicts the electrokinetic behavior of the particle. For the rest of the paper, we focus on calculating this factor for a semiconducting sphere in the electrolyte. Notice that we do not take into account any induced charge electroosmotic (ICEO) flow, which might affect the dielectrophoretic behavior at low frequencies of the applied field. However, experiments with metal spheres show that the influence of ICEO flow is small [7], and thus, the DEP motion is mainly driven by the forces on the induced particle dipole.

### 2.1. Particle Polarizability

We look for the polarizability of the system described above, and therefore, we will find the solution of the electric potential in our particle-electrolyte system and extract the polarizability from the dipolar term. We assume that the particle-electrolyte system is subjected to a homogeneous AC electric field along the Z-axes with magnitude $E_{0}$. Thus, the potential far from the particle can be written in spherical coordinates as ϕ(r→∞)=−Re[E0rcosθexp(iωt)]. The electric potential in the liquid ($\phi_{1}$) and the concentrations of positive ($c^{+}$) and negative ($c^{-}$) ions are governed by the PNP (Poisson–Nernst–Planck) equations [33]:

(7) $$\begin{array}{ccc} -\varepsilon_{1}\nabla^{2}\phi_{1} & = & e(c^{+}-c^{-}) \end{array}$$

(8) $$\begin{array}{ccc} \frac{\partial c^{+}}{\partial t} & = & -\nabla \cdot F^{+};\;\mathrm{with}\;F^{+}=-\mu^{+}c^{+}\nabla \phi_{1}-D^{+}\nabla c^{+}+c^{+}v \end{array}$$

(9) $$\begin{array}{ccc} \frac{\partial c^{-}}{\partial t} & = & -\nabla \cdot F^{-};\;\mathrm{with}\;F^{-}=\mu^{-}c^{-}\nabla \phi_{1}-D^{-}\nabla c^{-}+c^{-}v \end{array}$$

where $\varepsilon_{1}$ is the dielectric constant of the liquid medium and *e* is the proton charge. $D^{+}$, $\mu^{+}$ and $D^{-}$, $\mu^{-}$ are the diffusivities and mobilities of, respectively, positive and negative ions. v is the velocity field in the liquid. For simplicity, we assume an aqueous symmetric 1:1 electrolyte such as, for example, KCl. In this case, $\mu^{+}=\mu^{-}=\mu_{1}$ and $D^{+}=D^{-}=D_{1}$. The diffusivity and mobility of the ions are related by the Einstein–Smoluchowski relation $D/\mu =k_{B}T/e$.

Charge carriers in the semiconducting particle are electrons and holes. Following [34], the electric potential in the particle ($\phi_{2}$) and the concentrations of holes (*p*) and electrons (*n*) satisfy the following transport equations:

(10) $$\begin{array}{ccc} -\varepsilon_{2}\nabla^{2}\phi_{2} & = & e(p-n) \end{array}$$

(11) $$\begin{array}{ccc} \frac{\partial p}{\partial t}+\nabla \cdot F^{p} & = & r_{p};\;\mathrm{with}\;F^{p}=-\mu^{p}p\nabla \phi_{2}-D^{p}\nabla p \end{array}$$

(12) $$\begin{array}{ccc} \frac{\partial n}{\partial t}+\nabla \cdot F^{n} & = & r_{n};\;\mathrm{with}\;F^{n}=\mu^{n}n\nabla \phi_{2}-D^{n}\nabla n \end{array}$$

where $\varepsilon_{2}$ is the dielectric constant of the semiconducting particle. $D^{p}$, $\mu^{p}$ and $D^{n}$, $\mu^{n}$ are the diffusivities and mobilities of, respectively, holes and electrons. $r_{p}$ and $r_{n}$ are source terms that account for both the generation and recombination of charge carriers: $r_{p}=r_{n}=k_{1}n_{c}-k_{2}pn$, where $k_{1}$ and $k_{2}$ are constants and $n_{c}$ is the concentration of neutral centers. $n_{c}$ is homogeneous before any dissociation occurs, and for weak dissociation, it will remain nearly constant. Thus, no equation for $n_{c}$ is included. As we have assumed for ions in the electrolyte, we will consider that holes and electrons have the same mobilities and, according to Einstein–Smoluchowski relation, the same diffusivities: $\mu^{p}=\mu^{n}=\mu_{2}$ and $D^{p}=D^{n}=D_{2}$.

Since we are interested in the linear response (polarizability) of the particle–electrolyte system, we will assume that the applied signals are small and Equations (7) and (10) can be linearized. Under this assumption, and considering that the charge density in the electrolyte (medium j=1) is $\rho_{1}=e(c^{+}-c^{-}$), while in the particle (medium j=2) is $\rho_{2}=e(p-n$), the following equations apply [26,29]:

(13) $$\begin{array}{ccc} \nabla^{2}\phi_{j} & = & -\frac{\rho_{j}}{\varepsilon_{j}} \end{array}$$

(14) $$\begin{array}{ccc} D_{j}\nabla^{2}\rho_{j} & = & \left(\frac{\partial }{\partial t}+\frac{\sigma_{j}}{\varepsilon_{j}}\right)\rho_{j} \end{array}$$

where j=1,2. $\sigma_{j}$ is the electrical conductivity in medium *j*: $\sigma_{1}=e\mu_{1}\left(c_{0}^{+}+c_{0}^{-}\right)$ and $\sigma_{2}=e\mu_{2}\left(p_{0}+n_{0}\right)$, with $c_{0}^{+}$ and $c_{0}^{-}$ indicating the bulk concentrations of positive and negative ions, while $p_{0}$ and $n_{0}$ are the bulk concentrations of holes and electrons.

Boundary conditions at the particle–electrolyte interface (r=a) are obtained by imposing that charge carriers do not penetrate into the other medium, i.e., electron-holes do not migrate into the liquid and ions do not migrate into the particle:

(15) $${\left[\sigma_{j}\frac{\partial \phi_{j}}{\partial r}+D_{j}\frac{\partial \rho_{j}}{\partial r}\right]}_{r=a}=0$$

Furthermore, the continuity of the electric potential and the conservation of the total electric current holds at the particle surface:

(16) $$\begin{array}{ccc} \phi_{1}\left(r=a\right) & = & \phi_{2}\left(r=a\right); \end{array}$$

(17) $$\begin{array}{ccc} {\left.\varepsilon_{1}\frac{\partial \phi_{1}}{\partial r}\right|}_{r=a} & = & {\left.\varepsilon_{2}\frac{\partial \phi_{2}}{\partial r}\right|}_{r=a} \end{array}$$

note that the conservation of the total current reduces to the conservation of the displacement current since no charge carriers are allowed through the interface.

Taking into account the symmetry in our problem, it is convenient to define the following phasors for the electric potential ${\tilde{\phi }}_{j}\left(r\right)$ and charge density ${\tilde{\rho }}_{j}\left(r\right)$:

(18) ϕ(r,θ)=Re[ϕ˜(r)cosθexp(iωt)]

(19) ρ(r,θ)=Re[ρ˜(r)cosθexp(iωt)]

After applying Equations (13) and (14), the phasors satisfy the following equations:

(20) $$\begin{array}{ccc} D_{r}^{2}{\tilde{\phi }}_{j}\left(r\right) & = & -\frac{{\tilde{\rho }}_{j}}{\varepsilon_{j}} \end{array}$$

(21) $$\begin{array}{ccc} D_{r}^{2}{\tilde{\rho }}_{j}\left(r\right) & = & \left(i\omega +\frac{\sigma_{j}}{\varepsilon_{j}}\right)\frac{{\tilde{\rho }}_{j}}{D_{j}} \end{array}$$

where the following linear differential operator is used $D_{r}^{2}=\frac{d^{2}}{dr^{2}}+\frac{2}{r}\frac{d}{dr}-\frac{2}{r^{2}}$. We make use of non-dimensional quantities by introducing the following physical scales: the thermal voltage for the electric potential, $k_{B}T/e$; $2ec_{0j}$ for the charge density; and distances are scaled with the sphere radius *a*. The nondimensional potentials $\bar{\phi_{j}}=\tilde{\phi_{j}}e/k_{B}T$ and charge densities $\bar{\rho_{j}}=\tilde{\rho_{j}}/2ec_{j0}$ satisfy:

(22) $$\begin{array}{ccc} D_{\bar{r}}^{2}{\bar{\phi }}_{j}\left(\bar{r}\right) & = & -\frac{{\bar{\rho }}_{j}}{{\bar{\lambda }}_{Dj}^{2}} \end{array}$$

(23) $$\begin{array}{ccc} D_{\bar{r}}^{2}{\bar{\rho }}_{j}\left(\bar{r}\right) & = & \kappa_{j}^{2}{\bar{\rho }}_{j} \end{array}$$

where we have introduced the nondimensional Debye length in medium *j* as ${\bar{\lambda }}_{Dj}=\lambda_{Dj}/a$, with $\lambda_{Dj}=\sqrt{\left(\varepsilon_{j}k_{B}T\right)/\left(2e^{2}c_{j}^{0}\right)}$. The frequency-dependent factor $\kappa_{j}$ is given by $\kappa_{j}^{2}=\left(1+i\omega \varepsilon_{j}/\sigma_{j}\right)\left({\bar{\lambda }}_{Dj}^{2}\right)$.

Taking into account the boundary conditions far from the particle, the solutions for Equations (22) and (23) on the liquid side can be expressed as follows:

(24) $$\begin{array}{ccc} {\bar{\rho }}_{1}\left(\bar{r}\right) & = & \frac{A}{{\bar{r}}^{2}}\left(1+\kappa_{1}\bar{r}\right)\exp\left(-\kappa_{1}\bar{r}\right) \end{array}$$

(25) $$\begin{array}{ccc} {\bar{\phi }}_{1}\left(\bar{r}\right) & = & -\frac{{\bar{\rho }}_{1}}{\kappa_{1}^{2}{\bar{\lambda }}_{D1}^{2}}+{\bar{\psi }}_{1} \end{array}$$

where ${\bar{\psi }}_{1}$ must satisfy $D_{\bar{r}}^{2}{\bar{\psi }}_{1}=0$ and ${\bar{\psi }}_{1}\left(\bar{r}\to \infty \right)=-\bar{r}\bar{E_{0}}$. Taking $\bar{E_{0}}=1$, ${\bar{\psi }}_{1}=-\bar{r}+B/{\bar{r}}^{2}$.

On the particle side, and considering that the charge density vanishes at $\bar{r}=0$, the solutions of Equations (22) and (23) are written as:

(26) $$\begin{array}{ccc} {\bar{\rho }}_{2}\left(\bar{r}\right) & = & \frac{C}{{\bar{r}}^{2}}\left[\exp\left(-\kappa_{2}\bar{r}\right)\left(1+\kappa_{2}\bar{r}\right)-\exp\left(\kappa_{2}\bar{r}\right)\left(1-\kappa_{2}\bar{r}\right)\right] \end{array}$$

(27) $$\begin{array}{ccc} {\bar{\phi }}_{2}\left(\bar{r}\right) & = & -\frac{{\bar{\rho }}_{2}}{\kappa_{2}^{2}{\bar{\lambda }}_{D2}^{2}}+{\bar{\psi }}_{2} \end{array}$$

where ${\bar{\psi }}_{2}$ must satisfy $D_{\bar{r}}^{2}{\bar{\psi }}_{2}=0$ and remain finite for $\bar{r}=0$. Thus, ${\bar{\psi }}_{2}=F\bar{r}$.

Integration constants A,B,C, and *F* can be determined from the boundary conditions at the particle surface $\bar{r}=1$. Since we are interested in the dipolar term of the electric potential in the liquid, we solved for *B*, which coincides with the Clausius–Mossotti factor $\tilde{K}\left(\omega \right)$. After a long algebraic manipulation, we obtained the following expression:

(28) $$\tilde{K}\left(\omega \right)=B=\frac{1-\kappa_{1}^{2}{\bar{\lambda }}_{D1}^{2}-\left(\varepsilon_{1}/\varepsilon_{2}\right)\left(1-\kappa_{2}^{2}{\bar{\lambda }}_{D2}^{2}-G\left(\kappa_{2}\right)\right)\left(\kappa_{1}^{2}{\bar{\lambda }}_{D1}^{2}/\kappa_{2}^{2}{\bar{\lambda }}_{D2}^{2}\right)+\left(1+\kappa_{1}\right)/\left(\kappa_{1}^{2}+2\kappa_{1}+2\right)}{1-\kappa_{1}^{2}{\bar{\lambda }}_{D1}^{2}+2\left(\varepsilon_{1}/\varepsilon_{2}\right)\left(1-\kappa_{2}^{2}{\bar{\lambda }}_{D2}^{2}-G\left(\kappa_{2}\right)\right)\left(\kappa_{1}^{2}{\bar{\lambda }}_{D1}^{2}/\kappa_{2}^{2}{\bar{\lambda }}_{D2}^{2}\right)-2\left(1+\kappa_{1}\right)/\left(\kappa_{1}^{2}+2\kappa_{1}+2\right)}$$

where we have used the following function:

(29) $$G\left(\kappa_{2}\right)=\frac{\exp\left(-\kappa_{2}\right)\left(1+\kappa_{2}\right)-\exp\left(\kappa_{2}\right)\left(1-\kappa_{2}\right)}{\exp\left(\kappa_{2}\right)\left(\kappa_{2}^{2}+2-2\kappa_{2}\right)-\exp\left(-\kappa_{2}\right)\left(\kappa_{2}^{2}+2+2\kappa_{2}\right)}$$

In the Results Section, we will use Expression (28) to evaluate the dielectrophoresis and electrorotation of semiconducting spheres.

### 2.2. Limit of Thin Electrical Double Layers

The system polarizability as given by Equation (28) is valid for any values of the two length scales in our physical problem: the sphere radius and the thickness of the electrical double layer (EDL) that is induced at the particle–electrolyte interface. For a semiconductor–electrolyte interface, the EDL can be described as the combination of two diffuse layers: one in the liquid side and the other in the solid particle. The thickness of the diffuse layers is given by the Debye length ($\lambda_{Dj}$), which decreases with the reciprocal of the square root of the charge carrier concentration, as shown above [35]. Typical values for the Debye length are 30 nm for an aqueous solution of KCl with a concentration $10^{-4}$ M; and 40 nm for silicon with a doping density of $10^{16}\;{\mathrm{cm}}^{-3}$ [36]. Thus, in many experimental situations where the sphere radius is larger than a few microns, the EDL thickness will be much smaller than the particle size (${\bar{\lambda }}_{Dj}\ll 1$).

The mathematical problem can be greatly simplified if the EDL is much smaller than the particle dimensions; this is known as the thin EDL limit. The bulk of the conductive medium (electrolyte or semiconductor) remains electroneutral, and the solution to the electric potential satisfies the Laplace equation ($\nabla^{2}\phi_{j}=0$) with specific boundary conditions that account for the formation of the EDL at the electrolyte–particle interface [37]:

(30) $$\begin{array}{ccc} \left(\sigma_{2}+i\omega \varepsilon_{2}\right)\frac{\partial \phi_{2}}{\partial r} & = & \left(\sigma_{1}+i\omega \varepsilon_{1}\right)\frac{\partial \phi_{1}}{\partial r} \end{array}$$

(31) $$\begin{array}{ccc} \phi_{2}-\phi_{1} & = & \sigma_{2}\frac{\partial \phi_{2}}{\partial r}{\tilde{Z}}_{2}+\sigma_{1}\frac{\partial \phi_{1}}{\partial r}{\tilde{Z}}_{1} \end{array}$$

Equation (30) corresponds to the conservation of the total current, and it is valid as long as tangential currents at the interface are negligible. Equation (31) accounts for the voltage drop due to the charge accumulation at the EDL, where ${\tilde{Z}}_{j}$ stands for an effective surface impedance due to the thin diffuse layer of medium *j*:

(32) $$\tilde{Z_{j}}=\frac{\lambda_{Dj}}{i\omega \varepsilon_{j}\sqrt{1+i\omega \varepsilon_{j}/\sigma_{j}}}$$

These boundary conditions for an electrolyte–semiconductor interface were found in [38] for the study of AC electrosmotic flows that might appear at these interfaces.

In order to obtain the system polarizability, we assume, as before, that the electric potential far from the particle is given by ϕ(r→∞)=Re[rE0cosθexp(iωt)], and the solutions to the Laplace equation in the liquid and the solid are, respectively:

(33) $$\begin{array}{ccc} \phi_{1} & = & -E_{0}r\cos\theta +\frac{A}{r^{2}}\cos\theta \end{array}$$

(34) $$\begin{array}{ccc} \phi_{2} & = & Br\cos\theta \end{array}$$

where *A* and *B* are integration constants to be determined after applying boundary Conditions (30) and (31). Taking into account that *A* and the Clausius–Mossotti factor are related by $\tilde{K}=A/\left(E_{0}a^{3}\right)$, we obtain:

(35) $$\tilde{K}\left(\omega \right)=\frac{1-H(\omega )}{1+2H(\omega )}$$

where we introduce the function H(ω) as:

(36) $$H\left(\omega \right)=\frac{\sigma_{1}+i\omega \varepsilon_{1}}{\sigma_{2}+i\omega \varepsilon_{2}}\left(1+\frac{\kappa_{2}^{-1}}{i\omega \varepsilon_{2}/\sigma_{2}}\right)+\left(\frac{\kappa_{1}^{-1}}{i\omega \varepsilon_{1}/\sigma_{1}}\right)$$

Note that, for high signal frequencies, H(ω) corresponds to the liquid to solid permittivity ratio, and thus, Equation (35) becomes the well-known Clausius–Mossotti factor for a dielectric sphere. In the next section, we will show that the values of Equation (28) coincide with those of Equation (35) for small values of ${\bar{\lambda }}_{D1}$ and ${\bar{\lambda }}_{D2}$.

## 3. Results

According to Equations (5) and (6), the dielectrophoretic (DEP) and electrorotation (ROT) velocities are determined by the real and imaginary parts of the Clausius–Mossotti factor, respectively. In this section, we evaluate the expressions for this factor for spheres of two relevant semiconductor materials commonly used in applications: zinc-oxide and doped silicon. Physical properties at 300 K for these two semiconductor materials are shown, respectively, in Table 1 and Table 2. The Debye length for doped silicon was obtained as $\lambda_{D2}=\sqrt{\left(\varepsilon_{2}k_{B}T\right)/\left(e^{2}N_{D}\right)}$, where $N_{D}$ is the net density of dopants. The conductivity of doped silicon with $N_{D}=10^{13}\;{\mathrm{cm}}^{-3}$ was taken from the literature, and for other dopant densities, the conductivity scaled linearly with $N_{D}$. We also include the properties of the KCl aqueous solution, which we assumed to have a conductivity of 1.5 mS/m. This conductivity corresponds to an ionic strength of $10^{-4}$ M, a typical value in electrokinetic experiments. The Debye length for this electrolyte is around 30 nm, the same value as that for ZnO. Thus, the thin EDL approximation must be valid in experiments with microspheres with diameters around 10 μm and larger. However, the Debye length for n-type silicon with a doping concentration as low as $N_{D}=10^{13}\;{\mathrm{cm}}^{-3}$ is 1.28 μm, and the thin EDL approximation cannot be rigorously applied to microspheres.

**Figure 2:**
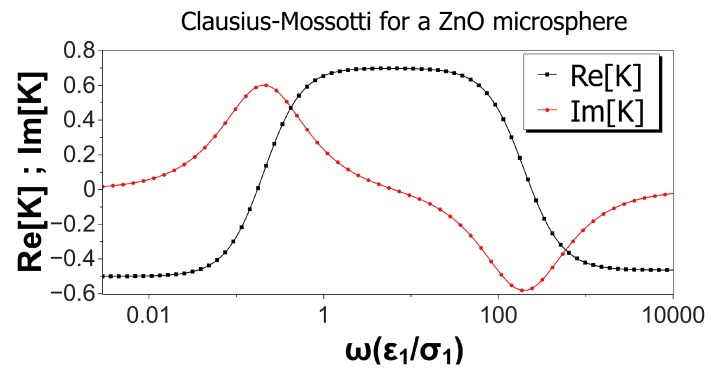
Real and imaginary parts of the Clausius–Mossotti factor versus nondimensional frequency for a ZnO microsphere immersed in a KCl solution. Values of the material properties are taken from Table 1. The microsphere is 10 microns in diameter, and the nondimensional Debye length is 0.006.

Figure 2 shows the real and imaginary parts of the Clausius–Mossotti factor versus nondimensional frequency for a ZnO microsphere immersed in a KCl solution. Values of the material properties are taken from Table 1, and the microsphere was 10 μm in diameter. Thus, the nondimensional Debye length was ${\bar{\lambda }}_{D1}={\bar{\lambda }}_{D2}=0.006$. Interestingly, the real part of *K* was negative for low and high frequencies, predicting negative DEP for these two limits, while positive DEP was expected for intermediate frequencies. According to the Kramers–König relations, an increasing value for the real part of *K* implies a positive peak for the imaginary part, while a decreasing value in Re[K] is accompanied by a negative peak in Im[K]. This is the result shown in Figure 2 for the imaginary part of the Clausius–Mossotti factor, predicting counterfield rotation at low frequencies and cofield for higher frequencies. As mentioned above, numerical values for the DEP velocity can be calculated from Expression (5). Using a typical experimental value for $|\nabla E_{0}^{2}|\approx 3\times 10^{12}\;V^{2}/m^{3}$ as in [7], we obtained DEP velocities ranging from −4.0 to 5.6 μm/s for the 10 μm ZnO microspheres. Likewise, using Expression (6) and a typical value of $E_{0}\approx 4$ kV/m, the counterfield peak angular velocity for these spheres was around 3.4 rad/s and slightly lower for the cofield peak velocity.

**Figure 3:**
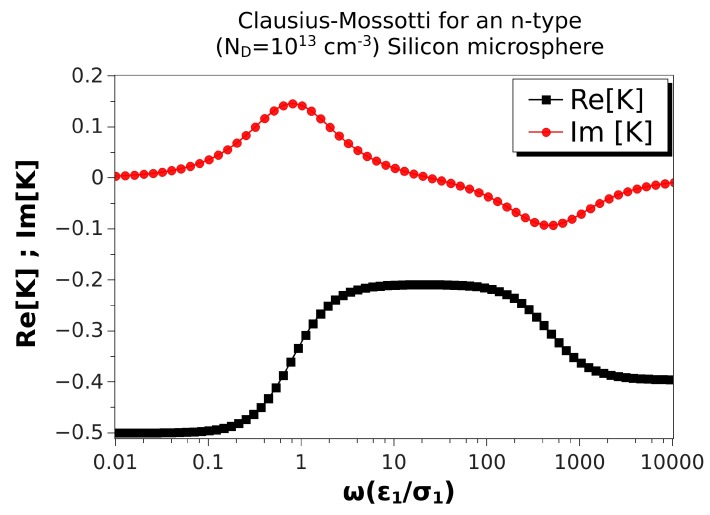
Real and imaginary parts of the Clausius–Mossotti factor versus nondimensional frequency for an n-type silicon microsphere immersed in a KCl solution. Values of the material properties are taken from Table 2. The microsphere is 10 μm in diameter. Thus, the nondimensional Debye length is ${\bar{\lambda }}_{D2}=0.26$.

Figure 3 shows the real and imaginary parts of the Clausius–Mossotti factor versus nondimensional frequency for an n-type silicon microsphere immersed in a KCl solution. Values of the material properties are taken from Table 2, and the microsphere was 10 μm in diameter. The doping density was $N_{D}=10^{13}\;{\mathrm{cm}}^{-3}$; the nondimensional Debye length for the solid side was ${\bar{\lambda }}_{D2}=0.26$. The real part did not change much with frequency, and it was always negative, though the two relaxations were still present. Consequently, the peaks in the imaginary part were smaller than in the case of ZnO (Figure 2). Thus, negative DEP of these microspheres was expected for all frequencies, with values between 4 and 1.6 μm/s for $|\nabla E_{0}^{2}|\approx 3\times 10^{12}\;V^{2}/m^{3}$. ROT velocities were relatively small in this case: using $E_{0}\approx 4$ kV/m, the peak of the counterfield angular velocity was 0.8 rad/s, while the cofield peak velocity was as low as 0.5 rad/s.

**Figure 4:**
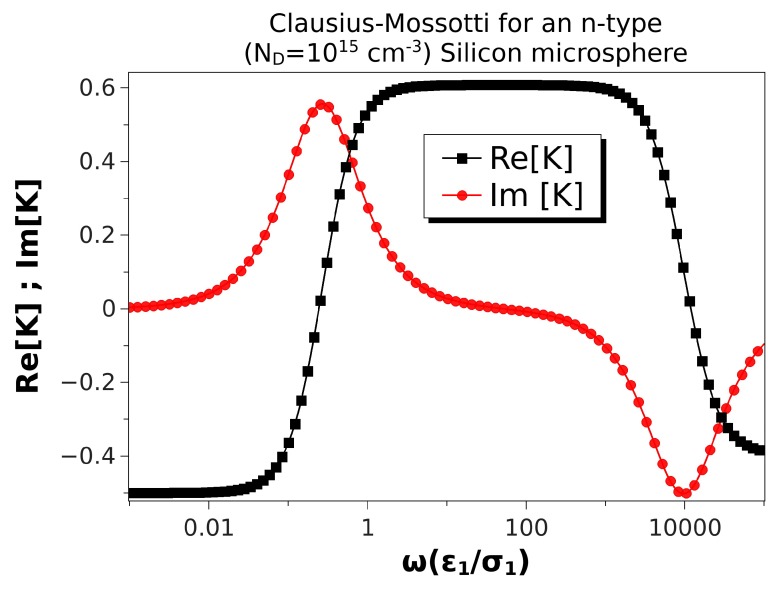
Real and imaginary parts of the Clausius–Mossotti factor versus nondimensional frequency for an n-type silicon microsphere immersed in a KCl solution. Particle conductivity is 22.6 S/m, and the Debye length is $\lambda_{D2}=0.331$ μm. The microsphere is 10 μm in diameter. Thus, the nondimensional Debye length is ${\bar{\lambda }}_{D2}=0.026$.

Figure 4 shows the Clausius–Mossotti factor versus nondimensional frequency for an n-type silicon microsphere with a higher doping concentration ($N_{D}=10^{15}\;{\mathrm{cm}}^{-3}$). In this case, the semiconductor conductivity was a hundred times larger than in Table 2, while the Debye length was ten times smaller than for $N_{D}=10^{13}\;{\mathrm{cm}}^{-3}$. The nondimensional Debye length for a microsphere of 10 μm in diameter was ${\bar{\lambda }}_{D2}=0.026$. In this situation, both positive and negative DEP were predicted again as in Figure 2. The only difference is that positive DEP was slightly smaller than that for a ZnO sphere because the Debye length was not as small as in that case. Correspondingly, the peaks of the Im[K] were also somewhat smaller than for ZnO.

### Comparisons with the Results for a Thin Electrical Double Layer

**Figure 5:**
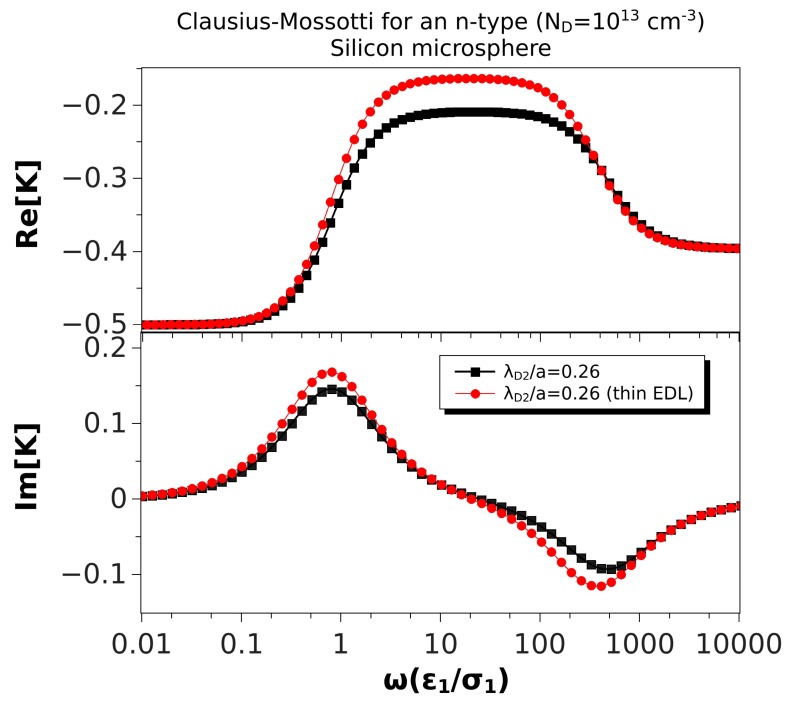
Real and imaginary parts of the Clausius–Mossotti factor versus nondimensional frequency for an n-type silicon ($N_{D}=10^{13}\;{\mathrm{cm}}^{-3}$) microsphere (10 μm) immersed in a KCl solution. The nondimensional Debye length in the particle is ${\bar{\lambda }}_{D2}=0.26$. The results of the thin EDL approximation (Equation (35)) are compared with the exact solution, Equation (28).

In this section, we compare the predictions of the thin double layer limit (Equation (35)) with the general expression of the Clausius–Mossotti factor (Equation (28)). As expected, the differences are only noticeable if the Debye length is not much smaller than the particle size. To explore this further, we will consider a semiconductor with properties as given in Table 2. The Debye length in this case was somewhat larger than 1 μm. We show in Figure 5 the Clausius–Mossotti factor obtained from Expressions (35) and (28) and for a microsphere of 10 μm in diameter, thus ${\bar{\lambda }}_{D2}=0.26$. We found that the relative difference between the maximum values of the real part was around 30%. The low frequency peaks of the imaginary part were relatively close (16% difference), while the high frequency peaks were off by 25%.

**Figure 6:**
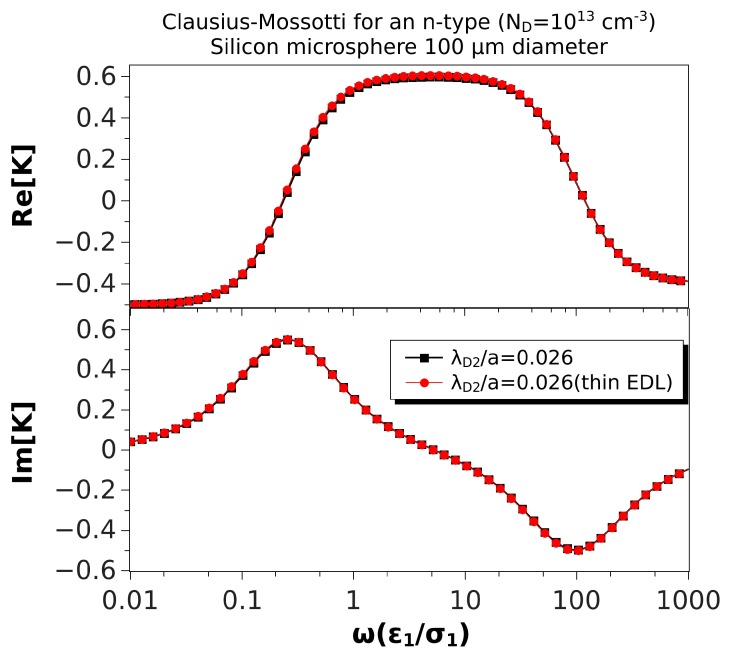
Real and imaginary parts of the Clausius–Mossotti factor versus nondimensional frequency for an n-type silicon ($N_{D}=10^{13}\;{\mathrm{cm}}^{-3}$) microsphere (100 μm) immersed in a KCl solution. The nondimensional Debye length in the particle is ${\bar{\lambda }}_{D2}=0.026$. The results of the thin EDL approximation (Equation (35)) are compared with the exact solution, Equation (28).

**Figure 7:**
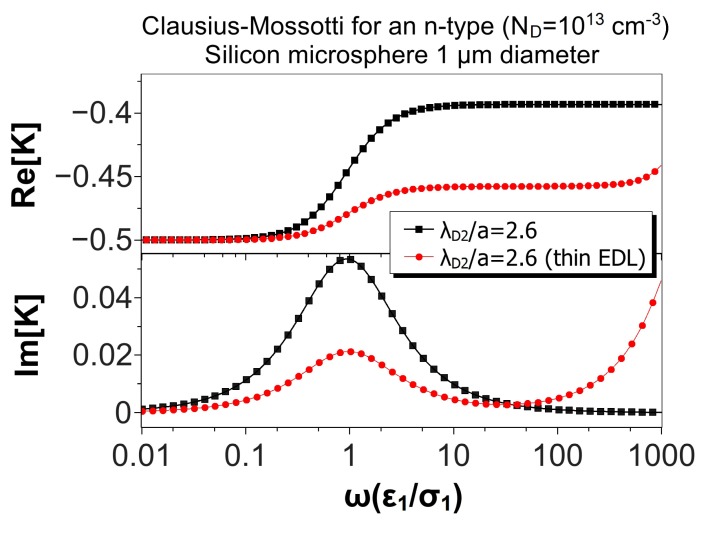
Real and imaginary parts of the Clausius–Mossotti factor versus nondimensional frequency for an n-type silicon ($N_{D}=10^{13}\;{\mathrm{cm}}^{-3}$) microsphere (1 μm) immersed in a KCl solution. The nondimensional Debye length in the particle is ${\bar{\lambda }}_{D2}=2.6$. The results of the thin EDL approximation (Equation (35)) are compared with the exact solution, Equation (28).

Figure 6 shows the same comparison, but for a microsphere of 100 μm in diameter. In this case, ${\bar{\lambda }}_{D2}=0.026$, and the thin EDL approximation looks very good. The relative difference between the maxima of the real parts was 1.2%, while the difference for the low and high frequency peaks of the imaginary parts were 0.7%. Finally, Figure 7 shows the results for a microsphere of 1 μm in diameter (${\bar{\lambda }}_{D2}=2.6$). As expected, the predictions of the thin EDL approximation were far from the exact theory.

## 4. Conclusions

**Figure 8:**
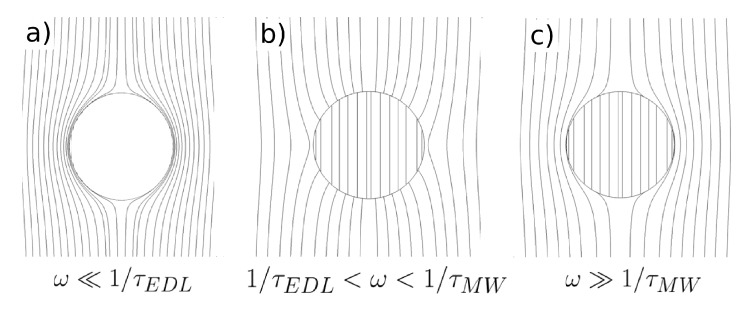
Electric field lines for a semiconducting particle immersed in an electrolyte: (**a**) Signal frequencies much smaller than the reciprocal of the double layer charging time ($1/\tau_{EDL}$); (**b**) intermediate frequencies; (**c**) frequencies much higher than the reciprocal of the Maxwell–Wagner characteristic time ($1/\tau_{MW}$).

We have shown the simplest analytical model that describes the electrokinetic behavior of a semiconducting sphere immersed in an aqueous electrolyte. The general behavior that we found is as follows:

- The model predicts negative dielectrophoresis at very low frequencies: the Clausius–Mossotti factor with real part equal to −0.5. This is the minimum value attainable for the Clausius–Mossotti factor of a sphere, and it arises when the electric field in the liquid surrounds the particle, as if the sphere were a perfectly insulating material; see Figure 8a. The reason for this is that the electrical double layer (EDL) is fully charged.
- As the signal frequency increases, there is not sufficient time for complete charging of the EDL by the electrical currents coming from the liquid bulk. The real part of the Clausius–Mossotti factor increases, leading to a weaker negative DEP and, eventually, to positive DEP behavior if the sphere conductivity is high enough; see Figure 8b.
- The Clausius–Mossotti factor shows a new relaxation for higher frequencies of the AC field; and the value of its real part decreases until a negative value that depends on the ratio of solid to liquid dielectric constants. This is the well-known Maxwell–Wagner relaxation, and negative dielectrophoresis occurs since the permittivity for the liquid is higher than for the solid; see Figure 8c.
- The imaginary part of the Clausius–Mossotti factor mirrors the behavior of the real part. As mentioned above, the EDL relaxation leads to an increase in the value of Re[K] with frequency, and consequently, a positive peak for the imaginary part appears. This peak corresponds to a prediction of counterfield electrorotation in experiments. Likewise, the decrease of the real part at higher frequencies predicts a cofield electrorotation.

We have also calculated the sphere polarizability assuming that the thickness of the EDL is much smaller than the particle size. This calculation is simpler than the full problem, and so is the expression for the Clausius–Mossotti factor obtained in this way. Evaluation of the theoretical expressions shows that the approximation yields acceptable results even if the particle is not much larger than the EDL thickness. Obviously, the thin double layer approximation cannot be used for particles of the same size or larger than the thickness of the EDL.

The values of the permittivities for the electrolyte and the semiconductor are taken as real numbers. This is a very good approximation for frequencies below the microwave region, i.e., below GHz. Interestingly, the maximum angular frequency in Figure 2, Figure 3, Figure 4, Figure 5, Figure 6 and Figure 7 is 10,000-times the reciprocal of the charge relaxation time in the electrolyte, $\sigma_{1}/\varepsilon_{1}$, i.e., around 2.1 MHz for an aqueous electrolyte with 1.5 mS/m. This means that our theoretical model is evaluated for a maximum dimensional frequency of around 3.4 GHz, already in the microwave region. Thus, the imaginary part of the permittivity could have some influence within the high-frequency range of the Clausius–Mossotti factor, but we do not expect any qualitative difference with the present results.

One important assumption of our theoretical modeling is that, for each media, we consider that positive and negative charge carriers have the same mobility. This could be a good approximation for intrinsic semiconductors, while differences may appear in doped semiconductors where the electrical conductivity is dominated by only one type of charge carrier, i.e., impurities remain immobile. Future work will be focused on the effect of the mobility asymmetry in the electrokinetic behavior of semiconductor spheres.

Finally, we did not consider the influence of induced charge electroosmosis (ICEO) [30] in the dielectrophoresis and electrorotation of the semiconductor spheres. It is well known that the ROT velocity is not affected by the ICEO flow if the Debye length in the electrolyte is small [29], as we assumed in our calculations. On the other hand, the ICEO flow might influence the particle displacement originated by a nonuniform field. The particle motion that arises as a combination of DEP and ICEO is known as dipolophoresis [26,27,40], and theoretically, the two mechanisms play an important role. However, experiments show that ICEO is much smaller than in theory due to several effects such as counterion crowding [41], surface roughness [42], and electrochemical reactions [43,44]. Thus, the main mechanism behind the displacement of microspheres is the action of the electric field on the induced dipole [7], i.e., DEP.

## Figures and Tables

**Table 1:** Values for the physical properties of the liquid and the semiconductor (ZnO) sphere in Figure 2. Values for ZnO are within the range reported in [39].

—	$\lambda_{D}$	σ	$\varepsilon /\varepsilon_{0}$
Semiconductor (ZnO)	30 nm	480 mS/m	3.9
Electrolyte (KCl in water)	30 nm	1.5 mS/m	80

**Table 2:** Values for the physical properties of the liquid and the semiconductor (n-type silicon) sphere in Figure 3 ($N_{D}=10^{13}\;{\mathrm{cm}}^{-3}$). Data obtained from [36].

—	$\lambda_{D}$	σ	$\varepsilon /\varepsilon_{0}$
Semiconductor (n-type silicon)	1.28 μm	226 mS/m	11.9
Electrolyte (KCl in water)	30 nm	1.5 mS/m	80

**Figure A1:**
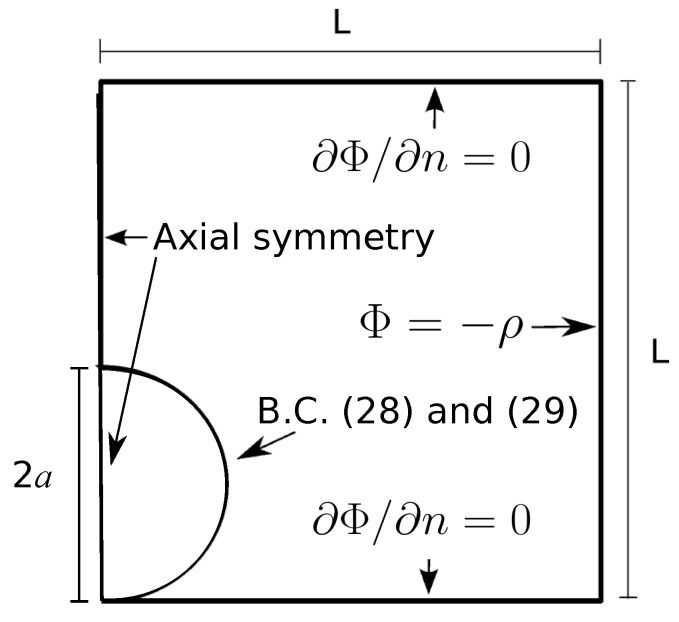
2D axisymmetric domain for computing the electrical torque on a sphere resting on a plane. The domain dimensions are much larger than the sphere radius L≫a (not to scale in the figure).

**Figure A2:**
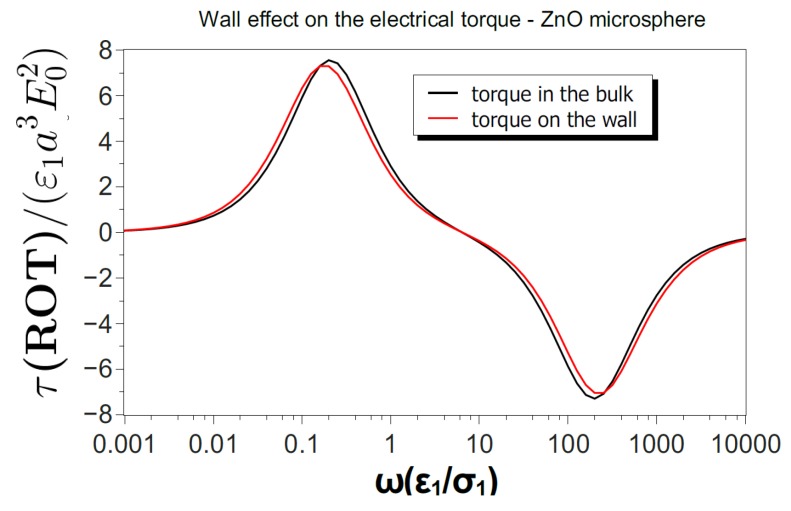
Time-averaged electrical torque (in units of $\varepsilon_{1}a^{3}E_{0}^{2}$) as a function of frequency exerted by a rotating electric field on a ZnO microsphere (${\bar{\lambda }}_{D1}={\bar{\lambda }}_{D2}=0.006$). The maximum torque for a sphere on the wall is 3.6% smaller than the maximum torque for the sphere in the liquid bulk.

## Appendix A. Wall Effects in the Electrorotation of a Semiconducting Sphere

Electrokinetics experiments with microparticles are usually carried out with microelectrode structures fabricated on a glass substrate. The microparticles are heavier than water and rest on the substrate after sedimentation. For future comparison with experimental data, we show in this section how to account for the influence of this wall on the electrorotation of semiconducting microspheres. In this analysis, we assume that the wall does not bear any electrical charge. The presence of an intrinsic surface charge could give rise to a more complicated wall-particle interaction and new phenomena beyond the scope of this work. We obtain that the viscous torque increases when the spheres are in contact with the glass substrate, while electrical torques are reduced.

In creeping flow conditions, the following expression corresponds to the viscous torque on a sphere near a wall that rotates around an axis perpendicular to the wall with angular velocity Ω [45]:

(A1) $$\tau_{\mathrm{viscous}}=-8\pi \eta a^{3}\zeta \left(3\right)\Omega$$

where ζ(x) is the Riemann zeta-function and ζ(3)=1.20206. By direct comparison with Equation (4), we find that the viscous torque is around 20% higher than for the same sphere in the liquid bulk.

The electrical torque on a body can be computed by integrating the Maxwell stress tensor (${\overset{↔}{T}}_{e}=\varepsilon EE-\left(1/2\right)\varepsilon E^{2}\overset{↔}{I}$) over the body surface *S*, $\tau_{e}=\left(1/2\right)\int_{S}\left(r\times {\overset{↔}{T}}_{e}\right)\times ndS$, where r is the position vector of a point on surface *S* and n is a unit vector normal to that surface. We are interested in the torque exerted on the particle by an electric field rotating in the horizontal plane. In this case, the electric potential far from the particle is written in cylindrical coordinates as ϕ(ρ→∞)=Re[−E0ρexp(i(ωt−φ))]. We assume that the thin EDL approximation is valid, and as in Section 2.2, the electric potential is the solution of the Laplace equation with boundary conditions on the particle surface given by (30) and (31). The solution of the electric potential can be written as ϕ(ρ,z,ϕ)=Re[Φ(ρ,z)exp(i(ωt−φ))], where we introduced the phasor Φ(ρ,z), which is the solution of the following equation:

(A2) $$\nabla^{2}\Phi \left(\rho ,z\right)-\Phi \left(\rho ,z\right)/\rho^{2}=0$$

We used COMSOL to find the solution of (A2) in the 2D axisymmetric domain shown in Figure A1. The time-averaged electrical torque is computed from the solution of Φ as:

(A3) τ(ROT)=ε12Im∫SΦ∂Φ*∂ndSuz

where *S* is the particle surface in Figure A1. Figure A2 shows the electrical torque in units of $\varepsilon_{1}a^{3}E_{0}^{2}$ as a function of frequency for a ZnO microsphere of 10 μm in diameter in two situations: the sphere in the liquid bulk and resting on the wall. The torque for the sphere in the bulk coincides with the torque predicted from Im[K] in Figure 1. The maximum electrical torque near the wall is 3.6% smaller than the maximum torque in the bulk.
